# Developing IoT Sensing System for Construction-Induced Vibration Monitoring and Impact Assessment

**DOI:** 10.3390/s20216120

**Published:** 2020-10-27

**Authors:** Qiuhan Meng, Songye Zhu

**Affiliations:** Department of Civil and Environmental Engineering, The Hong Kong Polytechnic University, Hong Kong, China; qiuhan.m.meng@connect.polyu.hk

**Keywords:** internet of things, cloud system, wireless sensor, construction-induced vibration monitoring, impact assessment

## Abstract

Construction activities often generate intensive ground-borne vibrations that may adversely affect structure safety, human comfort, and equipment functionality. Vibration monitoring systems are commonly deployed to assess the vibration impact on the surrounding environment during the construction period. However, traditional vibration monitoring systems are associated with limitations such as expensive devices, difficult installation, complex operation, etc. Few of these monitoring systems have integrated functions such as in situ data processing and remote data transmission and access. By leveraging the recent advances in information technology, an Internet of Things (IoT) sensing system has been developed to provide a promising alternative to the traditional vibration monitoring system. A microcomputer (Raspberry Pi) and a microelectromechanical systems (MEMS) accelerometer are adopted to minimize the system cost and size. A USB internet dongle is used to provide 4G communication with cloud. Time synchronization and different operation modes have been designed to achieve energy efficiency. The whole system is powered by a rechargeable solar battery, which completely avoids cabling work on construction sites. Various alarm functions, MySQL database for measurement data storage, and webpage-based user interface are built on a public cloud platform. The architecture of the IoT vibration sensing system and its working mechanism are introduced in detail. The performance of the developed IoT vibration sensing system has been successfully validated by a series of tests in the laboratory and on a selected construction site.

## 1. Introduction

**Figure 1:**
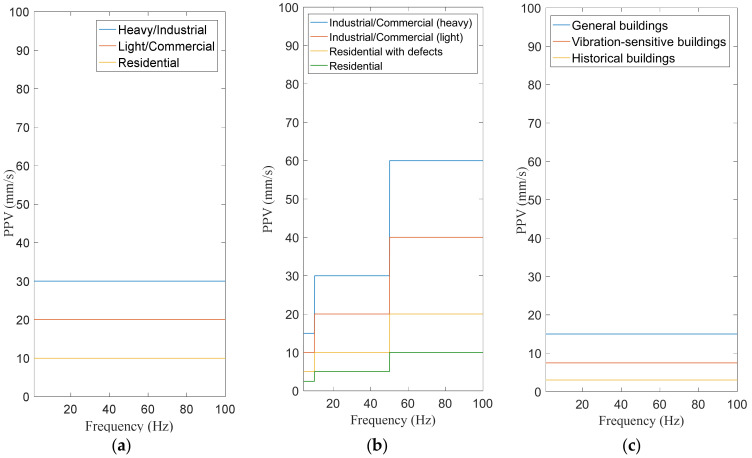
Maximum acceptable peak particle velocity (PPV) limits defined in (**a**) Eurocode 3 [10], (**b**) BS 5228 [9], and (**c**) Hong Kong Code [11] to protect various types of structures against structural damage.

Rapid urbanization leads to increasing population and building density in metropolitan cities (e.g., Hong Kong). Construction activities, such as blasting, demolition, piling, compaction, construction machinery, and so on, may expose a large number of nearby structures and facilities to intolerable levels of ground-borne vibrations. The effects of such construction-induced vibrations have been studied by researchers and practitioners for a long time [1,2,3,4,5,6,7]. A wide range of standards are also available to guide the evaluation of vibration impact on structures, facilities, and humans, including, but not limited to, British Standards [8,9], Eurocodes [10], International Organization for Standardization (ISO) [7], and other national and regional standards [11,12,13]. Three vibration indicators, namely, peak particle velocity (PPV), root mean square (RMS) velocity in the 1/3 octave band, and RMS acceleration in the 1/3 octave band, are commonly used to quantify the environmental effects of construction-induced vibrations in terms of structure safety, equipment functionality, and human comfort. PPV is the simplest indicator of the risk of structural damage, as particle velocity is proportional to induced strain during the passage of ground-borne waves [14]. In general, PPV attenuates quickly with increasing distance from a vibration source. Head and Jardine [15] presented an empirical attenuation formula in a power-law form. Figure 1 compares the vibration limits for different types of structures defined in Eurocode 3 [10], British Standard [9], and Hong Kong’s Code of Practice for Foundations [11]. Among them, only the British Standard [9] suggests frequency-dependent vibration limits. In Hong Kong, a limiting PPV of 15 mm/s is acceptable for buildings and major public utilities, while a more stringent PPV of 7.5 mm/s is required for protecting sensitive structures; for buildings of historical significance, the limiting PPV ranges from 2 to 3 mm/s [11]. 

On the other hand, many types of equipment and processes (such as scientific laboratories, hospital operating theaters, and microelectronic operation) are extremely sensitive to vibrations that are lower by orders of magnitude than those limits defined for structures. The indicator, RMS velocity in the 1/3 octave band spectrum, is widely adopted for vibration-sensitive equipment, in which each band covers a specific range of frequencies whose upper band frequency is the lower band frequency times the cube root of 2. ASHRAE [13] suggests the vibration limits based on the structural functions, which are 800, 400, 200, and 100 μm/s for workshops, offices, residential areas, and operation rooms, respectively. In addition, British Standard [9] provides a table of vibration limits concerning the vulnerability of facilities and equipment. Five categories containing specific types of equipment are given, and the corresponding RMS vibration velocities are taken from 50 to 3 μm/s in a frequency range from 4 to 80 Hz for transient vibration. For continuous vibrations, the allowable thresholds are only 40% of the permissible levels of intermittent vibrations. 

The RMS acceleration in the 1/3 octave band spectrum has been used in a baseline standard curve corresponding to the human perception level. Most standards provide the recommended threshold for human perception in different directions within the frequency range of 1–80 Hz. 

Considering construction-induced vibration as a non-negligible disturbance, a wide range of guidelines require compulsory monitoring processes during construction works to evaluate the potential impact of construction activities [12,16,17,18,19]. In general, vibration monitoring of ongoing construction activity can range from single-point measurements to simultaneous multi-point measurements. A simple monitoring operation takes occasional measurements at the concerned locations from time to time, while more advanced monitoring requires continuous measurement with real-time data processing, remote data display, and automatic alarm functions. Desirable characteristics of an advanced construction-induced monitoring system include: (1) easy installation and operation, (2) high sampling frequency, (3) embedded algorithms for vibration impact assessments concerning various vibration indicators, (4) real-time data processing capability, (5) graphical user interface that displays vibration impact assessment results to contractors. Given the highly specialized vibration monitoring devices, processes, and requirements, contractors often need to turn to third-party vibration consultants or specialists for help. Portable sensors and user-friendly monitoring system can considerably facilitate construction workers in their daily practice. The selection of an appropriate sensor should consider the needs of monitoring applications. For most construction-induced vibrations, a typical frequency range from 1 to 200 Hz is of interest. Therefore, a sampling rate greater than 400 Hz is preferred based on the Nyquist sampling theorem. The collected measurement results should be processed and compared with pre-specified vibration criteria continuously. Therefore, a construction-induced vibration monitoring system should have the capabilities of real-time data acquisition and processing, so that contractors can know vibration levels in real time and control the potential occurrence of negative impact in a timely manner.

Traditional tethered sensory systems have been successfully implemented to monitor many civil engineering structures in operational and extreme conditions [20,21,22,23]. However, their deployment is typically complicated and costly. Due to the maturity of microelectromechanical systems (MEMS) technology, wireless monitoring systems using MEMS sensors have been explored as a promising alternative to the wired system. MEMS-based wireless sensors integrate autonomous data acquisition, data processing algorithms, and wireless data transmission. Compared with wired systems, wireless sensors provide comparable functionality with considerably lower price, smaller size, and easier installation. Quite a few wireless sensor platforms have been developed and validated through tests in laboratories and real structures [24,25,26,27,28,29]. One of the earliest prototypes of the wireless modular monitoring system (WiMMA) integrating microcontroller unit (MCU) and wireless radio was developed by Straser et al. [24]. Later, a series of upgrading works, such as minimizing power consumption and embedding software, have been conducted [25,26]. The performance of WiMMA was validated by a shake table test with low-frequency excitations. Another representative wireless sensor platform was Imote2 from the Intel company. It features a low-power processor, large memory, better communication range, and high sampling frequency. The implementation of Imote2 requires external sensor boards; in addition to ITS 400 and ISM 400 from Intel, other customized sensor boards are also available. For instance, Rice and Spencer [27] developed the Structural Health Monitoring Accelerometer (SHM-A) board, which was mounted on an Imote2 and successfully applied to Jindo Bridge, Korea. To improve measurement quality and reduce noise, Jo et al. [28] used a uniaxial high-sensitivity accelerometer to develop the upgraded SHM-H board, which identified modal information from low-level vibration better than the case using the SHM-A board. Besides, Zou et al. [29] developed the improved single-sink multi-hop (ISSMH) board embedded on Imote2, which provided efficient data acquisition as proved by the laboratory experiments and field tests on Rainbow Bridge, Tokyo. Many additional successful applications in civil structures can be found in the literature [30,31,32,33,34,35]. 

By contrast, few researchers focused on vibration monitoring and vibration impact assessment at an on-going construction stage using wireless sensors, even though their portable size and easy installation features are very suitable for this purpose. One practical constraint is the sampling rate that is limited by the communication rate. A high sampling frequency is desirable to characterize impact-induced vibrations, but it may also cause data storage and transmission problems. For example, the SHM-A wireless sensor board supports the highest sampling rate of 280 Hz. But a considerably lower rate of 50 Hz was used for the Jindo Bridge implementation of Imote2 sensor nodes with SHM-A [27]. The Mica2 platform sampled single-axis vibration at a frequency of 250 Hz only for one minute because of the limited memory on sensor node [36]. The communication range associated with wireless sensors is another crucial issue. Well-known wireless communication protocols include IEEE 802.11, IEEE 802.15.1, and IEEE 802.15.4. In terms of transmission distance, a Wi-Fi (IEEE 802.11) access point often has a range of 20 m indoors while some advanced access points can cover up to a 250-m range outdoors [37]; the low power consumption limits transmission distances of Zigbee (IEEE 802.15.4) to 10–100 m depending on environmental conditions [38]; and the communication range of Bluetooth (IEEE 802.15.1) varies from 10 to 100 m [38], which is determined by the radio spectrum, transmit power, and receiver sensitivity [39]. Consequently, the data within a wireless sensor network cannot be transmitted in a very long distance due to the limitation of these transmission protocols. 

Nowadays, with the proposition of the concept of the Internet of Things (IoT) and the latest development of the next generation of networking, new opportunities have emerged by connecting wireless sensors to the Internet directly. IoT is a network of items (e.g., sensors) that can gather and exchange information through the Internet actively [40]. IoT enables easy access of data from a remote location in real time, which implies that a physical central station can be eliminated on a monitoring site. Other advantages include reduced operational costs and increased productivity. The development of IoT-based sensing system can provide an accurate, rapid, and cost-effective monitoring solution. In recent years, researchers have proved the probability of integrating wireless sensor systems into IoT for structural monitoring [41,42,43]. Some researchers also integrated IoT and cloud computing to achieve more intelligent data management and convenient data sharing [44,45,46].

To cater to the growing need in construction projects, this paper explores the feasibility and applicability of a novel IoT wireless sensing system for real-time construction-induced vibration monitoring and impact assessment. The proposed IoT sensing system uses a cheap and open-source microcomputer board, Raspberry Pi. The standardized design and highly expandability of the Raspberry Pi board allow for very flexible combinations with other hardware components (such as sensing and communication units) and thus can be easily customized for various applications, which can hardly be achieved by traditional wireless sensors with fixed designs. Each standalone wireless sensor node is recognized by the IoT platform, which eliminates cabling work and signal communication range limits. Convenient in situ installations and cheap prices of the hardware components will save financial and labor costs in practice. The integration of wireless sensors with IoT technology can realize real-time data transmission, impact assessment, and remote data access without distance limitation. The use of a cloud platform realizes flexible data storage and other specialized functions. 

In this paper, the hardware and software development of this system are presented in Section 2. Then, Section 3 illustrates the design of the cloud system. By establishing the sensing system and cloud system, Section 4 conducts the experimental study to evaluate the accuracy of the MEMS accelerometer by comparing the results with those from a traditional accelerometer, and its performance is validated by field measurements. Eventually, the major findings and conclusions are drawn in the last section.

## 2. Design of IoT Sensing System

**Figure 2:**
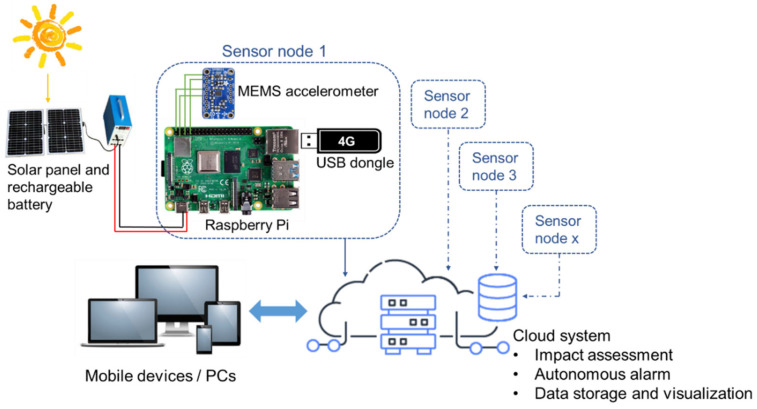
The structure of the Internet of Things (IoT) sensing system for construction vibration monitoring.

The proposed IoT sensing system for construction-induced vibration monitoring aims to use non-expensive hardware and general-purpose software to accommodate technological conditions in different measurement environments. To achieve this objective, an IoT sensing system prototype is developed by employing a Raspberry Pi board, a MEMS accelerometer sensor, a solar battery, and a 4G LTE Internet dongle, as well as specialized software that enables real-time workflow and other functions. As shown in Figure 2, the MEMS accelerometer is responsible for acquiring vibration data, while the Raspberry Pi is primarily responsible for analyzing data and transmitting data to the cloud for further data processing. Wireless transmission is achieved via 4G signal provided by a USB Internet dongle. Each wireless sensor node uses a standalone 4G signal to realize one-to-one communication with the cloud. Advanced functions, such as data storage, vibration impact assessment, alarming signals, user authorization, data sharing and visualization, are developed in the cloud system and can be customized for different sensor nodes. The whole system is powered by solar panels and rechargeable batteries. The key technical issues in the design of the IoT sensing system, including portable deployment, easy operation, restricted power supply, real-time data processing and assessment, and graphic display, are particularly addressed in this section.

### 2.1. Hardware System

#### 2.1.1. IoT Sensor Nodes

**Figure 3:**
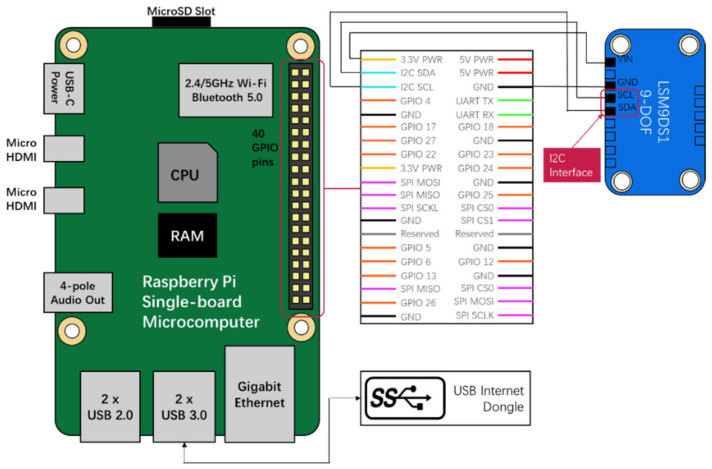
Schematic of IoT vibration sensor node using Raspberry Pi, microelectromechanical systems (MEMS) accelerometer, and 4G communication.

Figure 3 shows the configuration of a wireless vibration sensor node that is primarily responsible for measurement signal acquisition, data analysis, and data transmission to the cloud. Major components include a MEMS sensor, a single-board microcomputer (i.e., Raspberry Pi 4), a flash memory, and a USB 4G LTE Internet dongle. The LSM9DS1 MEMS sensor manufactured by STMicroelectronics company, which includes a triaxial accelerometer, a triaxial gyroscope, and a triaxial magnetometer, is employed as a vibration sensor. The LSM9DS1 is featured by low power consumption, high resolution (16-bit), and variable acceleration measurement ranges (±2 g, ±4 g, ±8 g, and ±16 g). Table 1 presents the corresponding acceleration resolution of the least significant bit (LSB). This device has different output data rates (ODRs), ranging from 14.9 to 952 Hz. The measurement results can be transferred through either Inter-Integrated Circuit (I2C) serial interface or Serial Peripheral Interface (SPI). The LSM9DS1 sensor may be powered by a 1.9–3.6 V power source and consumes 4.3 mA in a high-measuring mode.

Raspberry Pi is one of the most popular choices of open-source microcomputer, due to its small size, low cost, and full-featured nature, even compared with conventional PCs. The Raspberry Pi 4 model is used to acquire and process the acceleration data collected from the MEMS accelerometer. As a single-board microcomputer, Raspberry Pi contains an integrated central processing unit (CPU) and an on-chip graphics processing unit (GPU). Other provided functions include several USB ports, Gigabit Ethernet ports, USB Type-C for power supply, micro-HDMI for display, and audio and video ports. It also supports 2.4/5.0 GHz Wi-Fi and Bluetooth 5.0 connection. A variety of functions can be achieved by controlling general-purpose input/output (GPIO) pins of the Raspberry Pi through programming languages. Up to 40 GPIO pins offer the possibility of connecting multiple types of MEMS sensors to this platform and the adaptability to rapid sensor modifications. Besides, Raspberry Pi supports a Linux-based operating system (OS), which is associated with advantages in graphic interface, user-friendly programming language and communication. Compared with common MCUs (e.g., STM 32), the Raspberry Pi provides larger RAM and flash memory, enables multi-type sensing, more complicated data processing algorithms, and higher-level applications. There are multiple choices of programming languages including C, C++, PHP, Python, Ruby, and Visual Studio Code, as well as a variety of open-source libraries that offer versatile and efficient program development. Therefore, the Raspberry Pi is regarded as a cost-effective multi-function platform that can integrate signal processing, wireless communication, and other core functions. The compact size (85.6 mm × 56 mm × 21 mm) and low price (US $35) of the latest Raspberry Pi 4 model make it very portable and cost-effective, compared with traditional wireless sensors and DAQs. Other main hardware components include a flash memory for the installation of OS, drivers, software, and temporary data storage, and a USB 4G Internet dongle for wireless communication with the cloud platform.

**Figure 4:**
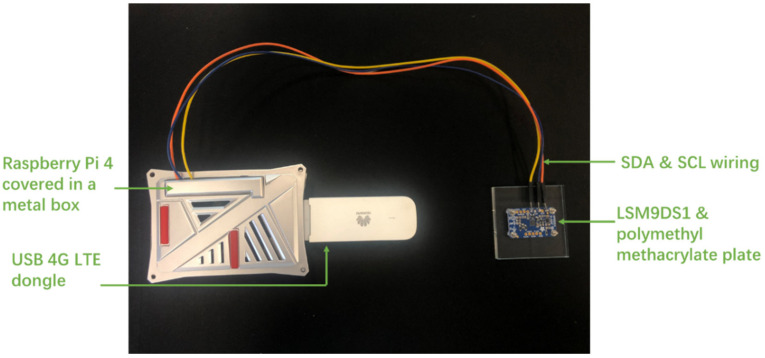
Fully assembled prototype of IoT sensing node.

In this study, the measured range of the LSM9DS1 accelerometer is set as ±2 g, corresponding to the highest resolution of 0.061 mg/LSB. The sampling rate is set as 952 Hz, which is regarded as sufficiently high to capture the high-frequency content of construction-induced vibrations. The LSM9DS1 accelerometer is powered by the 3.3 V power pin of the Raspberry Pi directly. The digitized measurement data are transferred to the Raspberry Pi board through a fast-mode I2C by connecting to Serial Data Line (SDA) pin (GPIO2) and Serial Clock Line (SCL) pin (GPIO3). Figure 4 shows a picture of a fully assembled sensor node prototype. The Raspberry Pi is packaged in a metal container to protect electronic components from harsh weather conditions. The MEMS accelerometer is screwed on a polymethyl methacrylate plate that can be conveniently attached to the surfaces of structures in practical applications.

#### 2.1.2. Wireless Communication

Transmission speed and reliability play important roles in wireless monitoring applications with real-time requirements. A significant time delay will jeopardize the quality of the vibration monitoring system. Moreover, a large amount of data will also cause network congestion. As discussed in the previous section, traditional wireless sensors commonly utilize Wi-Fi or ZigBee as transmission protocol. ZigBee has a relatively lower data transmission rate, while Wi-Fi relies on external router and power. To guarantee the reliability and effectiveness of wireless transmission, improving network performance and reducing the amount of transmitted data are both considered in this study. A USB dongle that connects to the fourth-generation (4G) mobile communication network is employed. The key parameters of the USB 4G dongle are summarized in Table 2. The 4G dongle can be directly powered by the USB 3.0 port of the Raspberry Pi board. This setting not only eliminates the limitation of communication range in wireless sensors, but also realizes independent point-to-point communication capability. It enables the flexible and standalone deployment of sensors that can satisfy general-purpose applications in a complex construction site environment. The selected 4G dongle also supports Wi-Fi sharing, which makes point-to-multipoint communication within a 100 m range possible. According to the laboratory and field tests, the effective data download and upload rates are around 38 and 8.5 Mbps, respectively, which depends on the selected 4G plan and in situ signal conditions. The testing results also indicated that such a data transmission rate is high enough to realize real-time triaxial measurement with a sampling frequency of 952 Hz without any data loss.

#### 2.1.3. Power Supply

Power supply is another critical issue for the wireless IoT sensing system. Using a power cable may compromise the advantages of wireless sensors. Since the 4G dongle and MEMS accelerometer are powered by the Raspberry Pi board, the overall power consumption of the Raspberry Pi board is measured by a USB current meter. The total active current of the IoT sensor node is around 700 mA, given a 5 V power supply. Among all the additional components, the USB 4G dongle consumes the greatest amount of power for wireless communication, and the average current is measured as 200 mA with 5 V voltage. Compared with other components, the power consumed by the MEMS sensor is small and nearly negligible. Therefore, a 12-V 20-Ah rechargeable battery is used as a power supply to the developed IoT sensor node, and the expected life of the battery is estimated to be 28.6 h in normal operating conditions. Solar panels with a total power of 50 W are used to charge the battery. When exposed to direct sunlight on the construction site, solar panels generate about 1.5 A current, which means that the battery can be fully charged in about 7 h. An average sunshine duration of 5 h/day in Hong Kong can keep the continuous operation of a vibration sensing node. 

Notably, the Raspberry Pi 4 is the latest model of the Raspberry series with significantly improved performance and increased power consumption. If lower power consumption is desirable in real applications, some older models in the Raspberry Pi series may be considered as an alternative. For example, the power consumption of the Raspberry Pi 2 B+ is only 1.3 W.

### 2.2. Software Development

#### 2.2.1. Operation Algorithm

**Figure 5:**
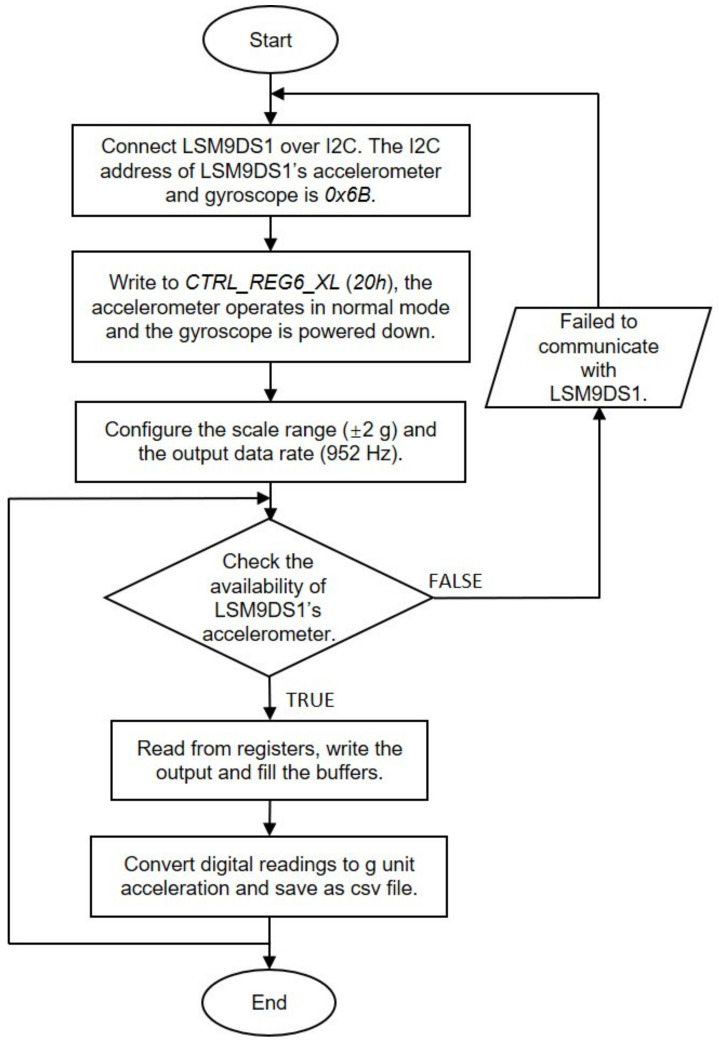
Flowchart of acceleration data acquisition and storage.

Figure 5 shows the flowchart of the data sensing, acquisition, and storage in the software system. The I2C bus is first instantiated at address 0×6B, and the operating mode is switched to Only Accelerometer Active. In this mode, the sampling frequency is set to 952 Hz by writing ODR_G [2: 0] = 110, and the linear acceleration range is set to ±2 g by writing FS_XL [1: 0] = 00. Table 3 provides a list of accelerometer registers embedded in the LSM9DS1, as well as the corresponding descriptions and addresses. If only the triaxial accelerometer is activated, multiple reads can be performed by starting from OUT_X_XL until OUT_Z_XL. Once OUT_Z_XL is read, the system automatically restarts from OUT_X_XL again. The value is expressed as a 16-bit word in two’s components (i.e., OUT_X_XL (28 h–29 h)). Subsequently, the measured data is converted to g value by multiplying the resolution. According to Table 1, the acceleration range of ±2 g corresponds to the resolution of 0.061 mg/LSB.

To save energy, two operation modes (namely, normal mode and low-frequency mode) and a trigger mechanism are designed. The normal mode works with 952 Hz ODR and real-time data processing and transmission during construction hours or daytime, while the low-frequency mode adopts lower sampling frequency without data transmission at night. The trigger mechanism is developed to reduce wireless communications, as well as processing overhead. When the system works at low-frequency mode and the measured value continuously exceeds the threshold over a period, the sensor operation will be transferred to the normal mode until the event finishes.

#### 2.2.2. Time Synchronization

In the developed IoT sensing system, each sensor node communicates with the cloud independently, and the communication among different nodes may not be available because of large separation distance and complex construction site environment. Therefore, the time of all the nodes needs to be synchronized with the cloud server instead of one another. Time synchronization through the Network Time Protocol (NTP) is adopted in the IoT sensing system. The NTP protocol estimates time drift between a hierarchy of clock references that are known as the stratum servers. The hierarchy starts at stratum 0, which represents high-precision clock source like atom clock, and goes down to less precise stratum 15. IoT devices with network connection can acquire clock time by polling the stratum server and estimate time drift based on the difference between the system clock and the remote reference clock. The Raspberry Pi 4 integrates the NTP service by default, which provides a convenient and low-cost option. Although the Global Position System (GPS)-based time synchronization provides higher precision than NTP, an extra GPS module and antenna need to be used, which increases both power consumption and hardware cost. The retail price of a GPS module is comparable to that of the Raspberry Pi board. 

**Figure 6:**
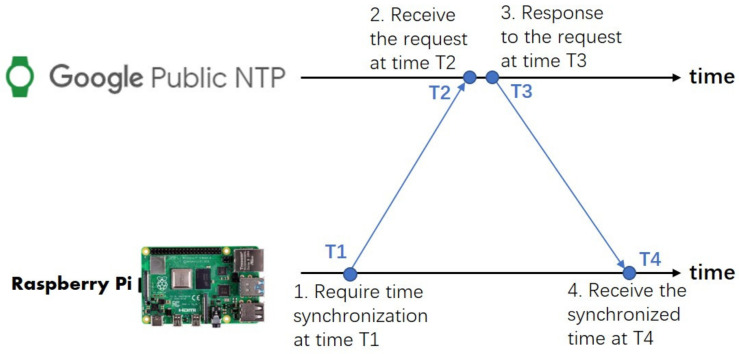
Network Time Protocol (NTP) synchronization procedure.

The synchronization rate depends on the stability of the clock hardware and the duration between subsequent measurements. The time drift is estimated by connecting the Raspberry Pi to the Internet by the 4G dongle. The Google NTP server (time.google.com) is used as the reference clock to calculate the time drift of the Raspberry Pi. Figure 6 illustrates the procedure of time synchronization, wherein the time drift can be calculated as:

(1) $$\mathrm{Time}\;\mathrm{drift}\;=\frac{\left(T_{2}-T_{1})+(T_{4}-T_{3}\right)}{2},$$

**Figure 7:**
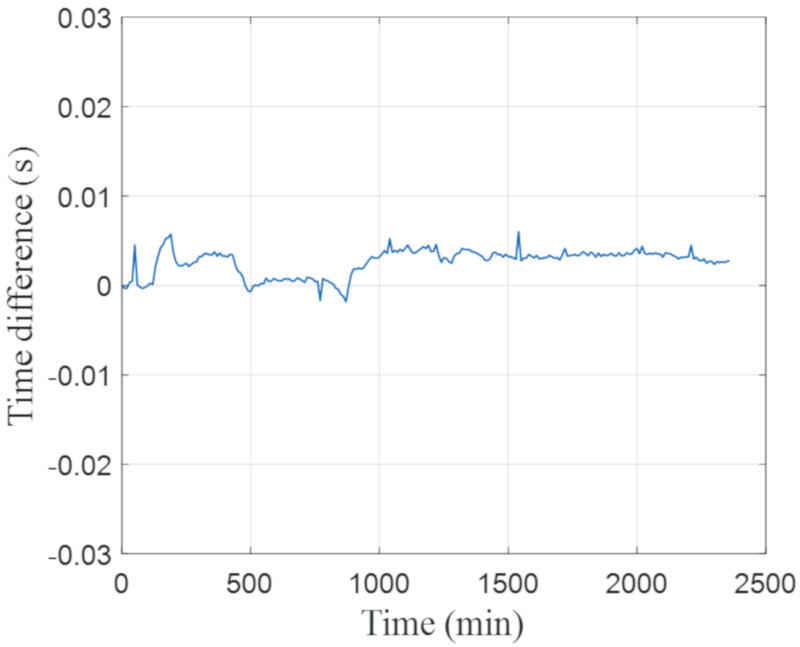
Time drift with Google NTP server.

Figure 7 presents the time drift between the Raspberry Pi and Google NTP server measured in the experiment, wherein the internal system clock was initially synchronized to the NTP server at the beginning and was then compared with the NTP server every minute during the 40-h experimental period. As shown in Figure 7, the absolute variability of time drift is less than 10 ms. The possible causes for time delay include sender processing delay, transmission time delay, and receiver processing delay. Therefore, the accuracy of the NTP-based time synchronization depends on the Internet status, and good network conditions can minimize transmission time delay. For construction-induced vibration impact assessment, vibration indexes (such as PPV and RMS velocity in the 1/3 octave band spectrum) are calculated over a specified duration (e.g., 5 s). Therefore, a time drift/difference of 10 ms can satisfy the measurement requirement and does not compromise the vibration assessment quality. 

The time synchronization strategy between the NTP server and Raspberry Pi clock is designed as follows: the time drift is examined every 5 min and the mean absolute time drift is calculated every hour; if the mean absolute time drift of this hour does not exceed the drift threshold (i.e., 10 ms), the clock adjustment is not necessary and the time drift will be recalculated in the next hour; if the mean absolute time drift is greater than 10 ms, the system clock will be adjusted according to the network time, and the adjustment will be recorded in a log file.

#### 2.2.3. Local Data Processing

**Figure 8:**
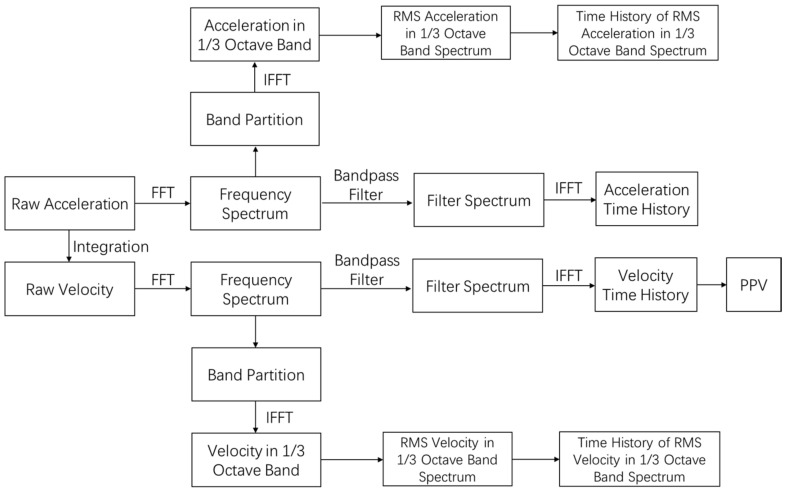
Flowchart of signal processing and data transmission.

With the upgraded CPU, the Raspberry Pi 4 provides an impressive computational capacity to conduct real-time continuous data analysis. Therefore, a local data processing algorithm is developed, and the corresponding software is embedded in the IoT sensing node. As demonstrated in Figure 8, a floating-point fast Fourier transform (FFT) together with bandpass filter is applied to the raw acceleration signals to remove noise and static components. Two vibration indicators, PPV and RMS velocity in the 1/3 octave band spectrum, are calculated to evaluate the impact on structure safety and sensitive equipment functionality. Another vibration indicator, RMS acceleration in the 1/3 octave band spectrum, is calculated to evaluate the human comfort level. The PPV is calculated by a built-in array max and min function, while the RMS velocity and RMS acceleration in the 1/3 octave band spectrum are calculated with the octave band partition and statistics function. The system computation power can support real-time data collection, processing, and storage, given the selected sampling frequency of 952 Hz. During the signal processing, the CPU temperature rises from 47 ºC to 56 ºC, and the CPU utilization increases from 2.35% to 28.91%. The frequency spectrum, time history data, and analyzed vibration indicators are stored in the flash memory temporarily. Given the sampling frequency, a CSV file containing raw acceleration data typically occupies 43 kb per second. A sufficient flash memory size should be selected to avoid data loss due to lack of space. In general, the Raspberry Pi 4 will transfer all these data simultaneously to cloud for permanent data storage and visualization.

## 3. Data Storage and Virtualization in Cloud

**Figure 9:**
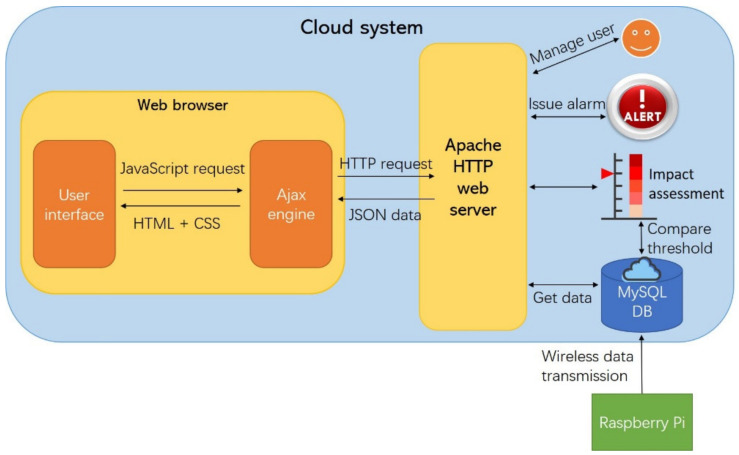
Cloud system architecture.

The public cloud environment offered by commercial providers has been used for the development of many applications and software, because of its advantages including scalability, easy-to-use, and high security. Users only pay for the services and capacity they need and access those services via the Internet. Ali Yun (a public cloud provider) is selected to build a cloud system for the IoT sensing system. Major functions of the cloud system, including data storage, vibration impact assessment, autonomous alarm, user access, data sharing and visualization, are shown in Figure 9. The Raspberry Pi is connected to the Internet via USB 4G dongle and transfer vibration measurements to the MySQL database by a program written in Python. The web application is built with Apache web server, and the client-end web interface is programmed in Preprocessor (PHP), Hyper Text Markup Language (HTML), Cascading Style Sheets (CSS), and JavaScript. Users can access the web interface from anywhere via Internet. A database of various vibration indicators and limits is established in the cloud system in advance, and vibration impact assessment is realized by comparing real-time vibration levels with relevant vibration limits. Autonomous alarm is sent to concerned parties in different formats (e.g., email and SMS message).

### 3.1. Cloud Database

Considering the relatively limited memory space provided by the Raspberry Pi, a MySQL database is built for long-term data storage. MySQL is an open-source database suitable for quick queries of structured data. All the raw and processed data, as well as analysis results are organized into one or more tables and stored in MySQL. For a long-term monitoring project, the measured data will be partitioned into different tables defined by date. Each table has a certain identity and consistent index, field type, and character set. Operations, such as create table, modify data, and extract data from the database, are realized by Structured Query Language (SQL). To keep tracking of these operations, three types of log files are created, namely, error log, query log, and binary log. The error log records all warning and error messages during the startup, standby, or shutdown of the servers. The general query log records SQL queries from client connections, which helps database optimization. The binary log writes down the information of processes and events regarding database modification, where the cloud will prevent malicious data modification. Moreover, all data will be backed up periodically in case of accidental data loss or damage.

In addition, authorized users can remotely configure measurement parameters (such as measurement duration or measurement index) by connecting to the Raspberry Pi through the Internet. MySQL can automatically respond to the changes in data caused by new configurations. For example, when different types of data are sent to MySQL, the corresponding table will be created with pre-defined table structures and parameters.

### 3.2. Web Interface

A webpage-based user interface is designed to show real-time construction-induced vibration impact assessment results, which can be accessed conveniently by mobile apps or web browsers through the Internet. The framework of the webpage is designed on a simplified Model-View (MV) pattern, in which the Model notifies the Views when the state of the data has changed, and the Views retrieve the new model state and render the latest state of the data. As depicted in Figure 9, the client-end web interface adopts HTML, CSS, JavaScript, Ajax, jQuery, and Highcharts. HTML and CSS are implemented together in the style of the webpage. JavaScript and jQuery are used to enable interactive communication between the cloud server and clients in JavaScript Object Notation (JSON) data format. Asynchronous JavaScript and XML, also known as Ajax, realizes the information transmission between webpage and web server asynchronously through XMLHttpRequest in the backend without interrupting the display of existing pages. Highcharts is a JavaScript charting library featuring attractive looks and interactive styles. It is used to create a wide variety of graphs for static or dynamic sensor data display. Highcharts is able to auto-refresh the chart following newly added data instead of reloading the entire graph. Ajax and jQery call continuous flow of data from the MySQL databases and serialized into JSON format to be displayed on the webpage. 

In the designed client-end webpage, several utilities for experimentation and demonstration purposes are also designed, including real-time display, data access, and system management. With real-time display utility, users can monitor the sensor data updates in real time. Also, historical data stored in the database can be downloaded or viewed both online and offline conveniently. The provided options enable to download the data as CSV files, print data image in PNG or JPEG format, and view data table. With system management utility, administrative users can configure information and privileges of different users. On the other hand, the webpage can be updated without interrupting the operation of sensor nodes or algorithms inside the Raspberry Pi, which makes it convenient to perform customization and revision of the display interface.

### 3.3. Vibration Impact Assessment and Autonomous Alarm

The vibration impact is assessed based on the real-time vibration levels and the database of vibration limits and displayed to users through graphic interfaces. An autonomous alarm is developed using built-in functions in the web interface. The pre-specified acceptable limits for the vibration indicators are set as threshold values. Upon the occurrence of the exceedance of vibration limits, a variety of alarming signals will be issued with limited time delay. By using the Email server software and SMTP email function, an immediate email notification that indicates the real-time vibration levels and the potentially affected structures/facilities will be sent to the contractors and other concerned parties. The visual and audio alarming signals are also designed in the user interface by using the built-in Boolean function and Beep function.

## 4. Validation through Laboratory and Field Tests

To prove the performance and accuracy of the proposed IoT sensing system, a series of validation tests were performed in the laboratory and on a construction site. The results obtained from both tests indicate that the developed MEMS accelerometer has satisfactory sensitivity and the IoT sensing system can collect, process, transmit, analyze, and display data accurately and reliably. This section presents the results obtained in the laboratory and field tests.

### 4.1. Calibration of MEMS Accelerometer

**Figure 10:**
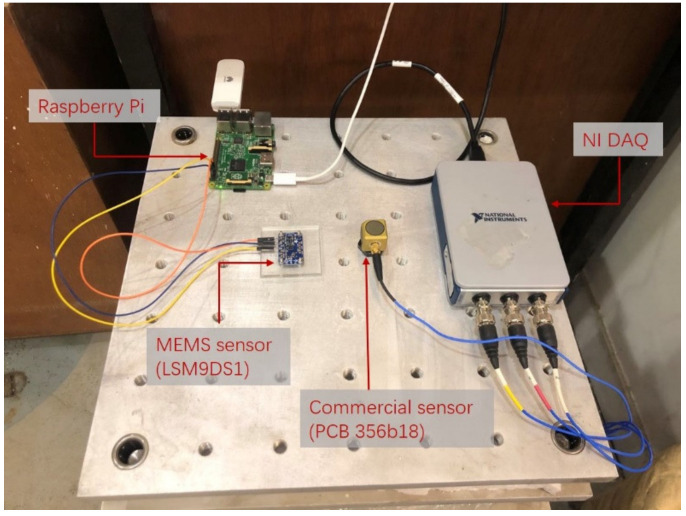
Shake table test setup.

The laboratory calibration tests were conducted on a small shake table (ASP 420), aiming to examine the accuracy of the selected MEMS accelerometer (LSM9DS1) in the vibration range of interest in construction projects. The measurement accuracy of the MEMS accelerometer was compared with another piezoelectric accelerometer (PCB 356b18) manufactured by PCB Piezotronics. The PCB accelerometer is an integrated-circuit-piezoelectric (ICP), high-sensitivity sensor with a sensitivity of 1000 mV/g and an output resolution of 24 bit. Figure 10 shows the experimental setup of the shake table tests. The MEMS accelerometer (LSM9DS1) was screwed on a polymethyl methacrylate plate, and the latter was attached to the shake table surface by epoxy resin glue. The LSM9DS1 accelerometer (configured to ±2 g range and 952 Hz ODR) was connected to the Raspberry Pi. The commercial accelerometer (PCB 356b18) was mounted on the same surface of the shake table and was connected to another commercial four-channel DAQ from National Instruments (cDAQ-9171). The sampling frequency of the commercial DAQ is set to 1000 Hz. Table 4 displays the comparison between the cost of the commercial system used in the experiment and the IoT sensing system. The approximate hardware cost of the commercial system is around 2620 US dollars; in contrast, the cost of the proposed IoT sensing system is only 70 US dollars.

A simple offset calibration was conducted before the formal tests to eliminate the inherent bias of the MEMS accelerometer. The constant gravity magnitude was used to calibrate triaxial measurement. For example, when the MEMS accelerometer was placed on a horizontal surface facing upwards and downwards, the *z*-axis reading should be +1 g and −1 g, respectively. The offset can be calculated by the following equation:

(2) $$Z_{offset}=\left(\frac{\sum Z_{up}}{n}+\frac{\sum Z_{down}}{n}\right)/2,$$

where *n* is the number of readings.

**Figure 11:**
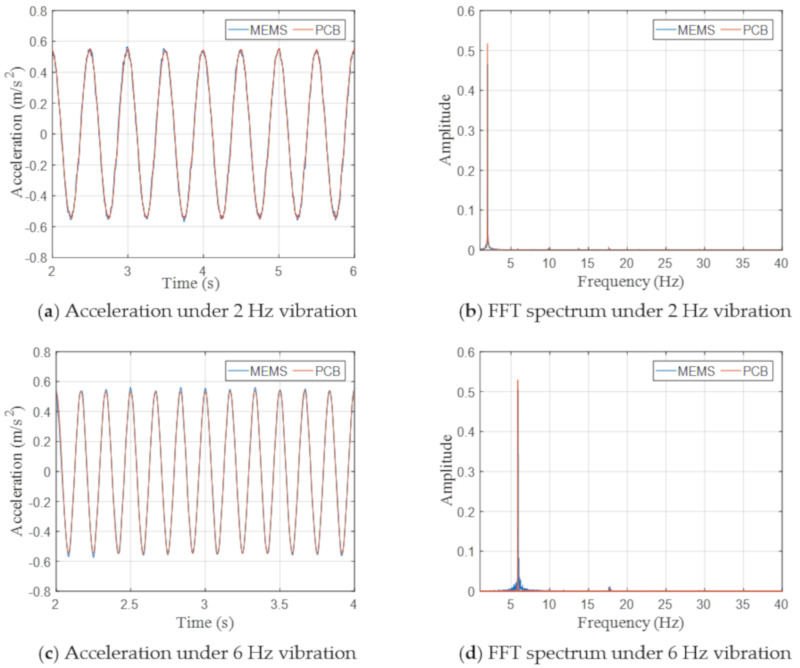

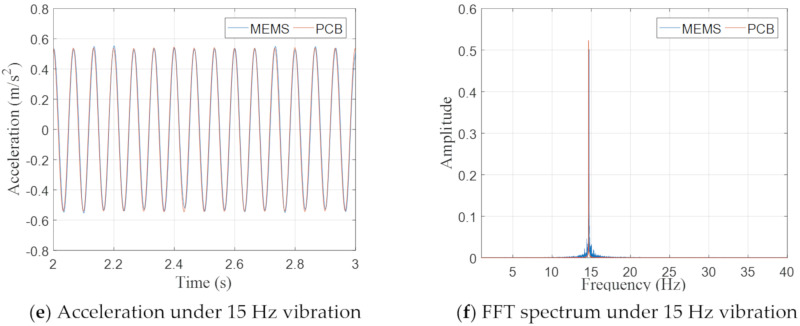
Measured acceleration time histories and fast Fourier transform (FFT) spectra by MEMS and PCB accelerometers with the excitation level 0.05 g.

The shake table generated harmonic vibrations at different frequencies (1, 2, 4, 6, 8, 15, and 20 Hz) and at different amplitude levels, which were measured by both accelerometers simultaneously. The test results indicate that the MEMS accelerometer showed satisfactory accuracy under the vibrations with the amplitude down to 0.01 g and the frequency down to 1 Hz, wherein the amplitude and frequency represent the lower limits of the testing capacity of the shake table and the signal generator (Keysight 33500B), instated of those of the MEMS accelerometer. Figure 11 shows the acceleration time histories and FFT spectra under the vibrations with the amplitude of 0.05 g and three representative frequencies. The accelerations measured by the MEMS accelerometer show good consistency with those by the PCB accelerometer in both time and frequency domain. 

The accuracy of the measured acceleration is further quantified via maximum peak error (PE) and root mean square error (RMSE), as presented in Equations (3) and (4), respectively. PE determines the accuracy of the peak measurement, and RMSE gives an average error percentage by comparing the observed values and reference values. PE and RMSE are defined as follows:

(3) $$\mathrm{PE}=\left|\frac{A-B}{A}\right|,$$

where *A* and *B* are the maximum absolute accelerations measured by the PCB accelerometer and MEMS accelerometer, respectively;

(4) $$\mathrm{RMSE}=\sqrt{\frac{\sum_{i=1}^{n}{\left(a_{i}-b_{i}\right)}^{2}}{N}},$$

where *a_i_* and *b_i_* are the values at the *i*-th time step from different types of accelerometers, and *N* is the total number of data points.

**Figure 12:**
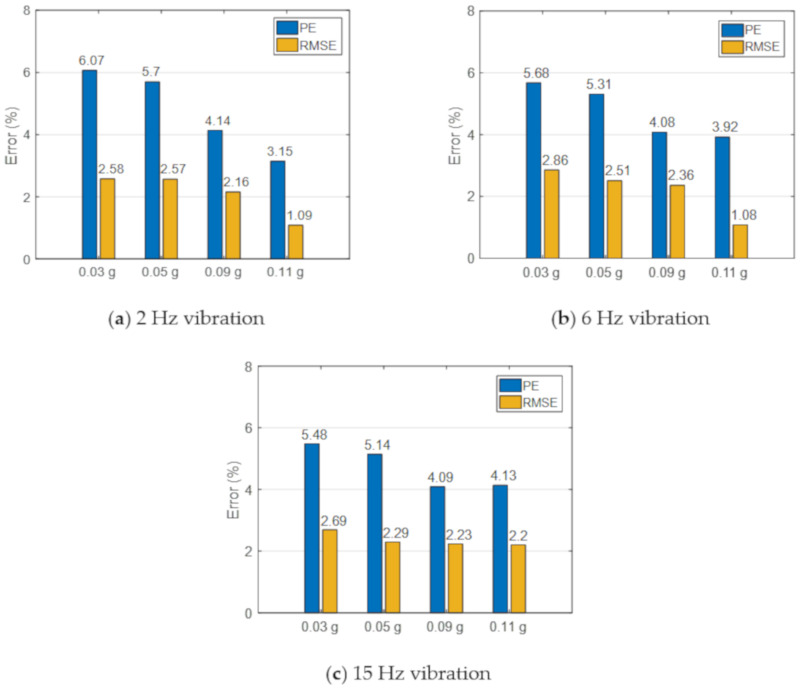
Peak error and root mean square (RMS) error of measured acceleration responses.

Statistic results of the PE and RMSE under the vibrations of three different frequencies (2, 6, and 15 Hz) and different amplitudes (0.03 g, 0.05 g, 0.09 g, and 0.11 g) are presented in Figure 12. In general, the PE decreases with the increasing vibration amplitude. The maximum difference in the peak acceleration is 6.07% at a low vibration amplitude, and most of the PEs are less than 5%. RMSEs are significantly lower than PEs, and all the RMSEs are smaller than 3%. 

**Figure 13:**
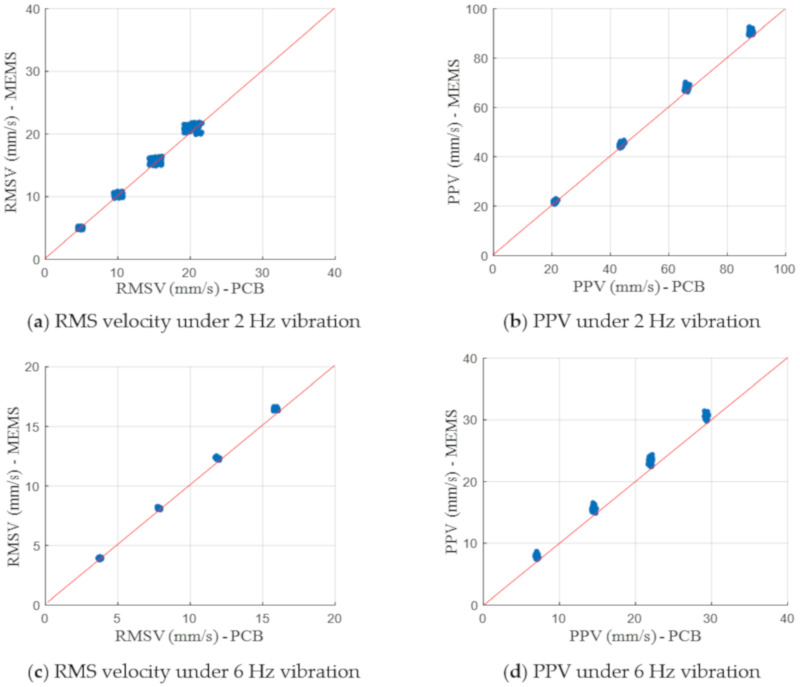

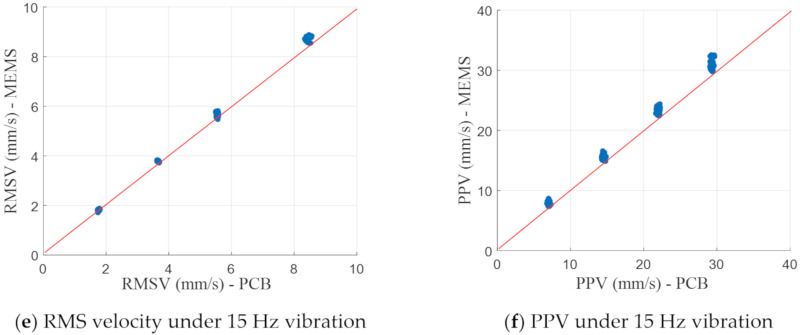
Comparison of PCB accelerometer and MEMS accelerometer in terms of RMS velocity in the 1/3 octave band and PPV.

In addition to the acceleration comparison, two velocity-based vibration indicators, namely, PPV and RMS velocity in the 1/3 octave band spectra, were also calculated and compared. Figure 13 shows the comparison of the PCB accelerometer and MEMS accelerometer with respect to these two vibration indicators that are commonly adopted for construction vibration impact assessment. The consistent results in the comparison indicate the satisfactory measurement accuracy of the adopted MEMS accelerometer and the data processing algorithm. Notably, the slight difference observed between two tested accelerometers may not be fully caused by the measurement errors or noise. Different installation methods and sampling frequencies of two accelerometers may also contribute to the observed deviations.

**Figure 14:**
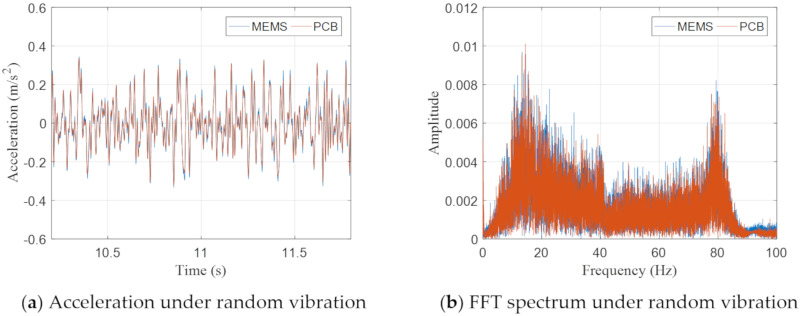
Measured acceleration time histories and FFT spectra by the MEMS and PCB accelerometers under random excitation.

The comparison between the MEMS and PCB accelerometers was also conducted under a band-limited (5–90 Hz) random excitation with the peak acceleration of around 0.04 g. As shown in Figure 14, the excellent agreement between the measurement results of two accelerometers proves the accuracy of the MEMS accelerometer in the measurement of random excitation within the frequency range of interest. The relative difference between the results of two sensors is only 2.35%

### 4.2. Field Experiments

**Figure 15:**
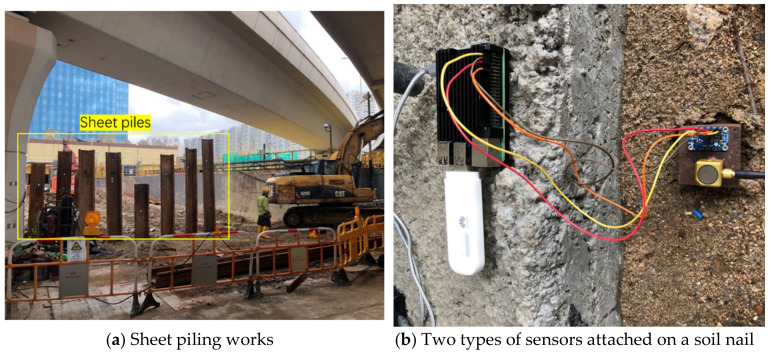
Field tests of the IoT sensing system on a selected construction site in Hong Kong.

To examine the performance of the IoT sensing system in real applications, field experiments were conducted on a selected construction site in Hong Kong with ongoing sheet piling works (as shown in Figure 15a). The Raspberry Pi was packaged in a metal box for protection. Besides the IoT sensing system, a commercial wired monitoring system including a triaxial accelerometer (PCB 356b18), a DAQ (cDAQ-9171), and a computer that were the same as the laboratory test, was also installed. The LabView software was used to digitalize and analyze the acceleration signals from the wired monitoring system. The sampling frequencies of the wired monitoring system and IoT sensing system were set to be 1000 and 952 Hz, respectively. To facilitate the comparison, the IoT sensing system and the wired monitoring system were deployed at the same locations, wherein the PCB 356b18 accelerometer and MEMS accelerometer LSM9DS1 were attached on top of the soil nail that directly plugged into the soil (as shown in Figure 15b). The measurements were carried out at different distances from the vibration source (i.e., the sheet piling location). During the entire testing process, the designed IoT sensing system worked smoothly without any problems such as data losses or communication failure.

**Figure 16:**
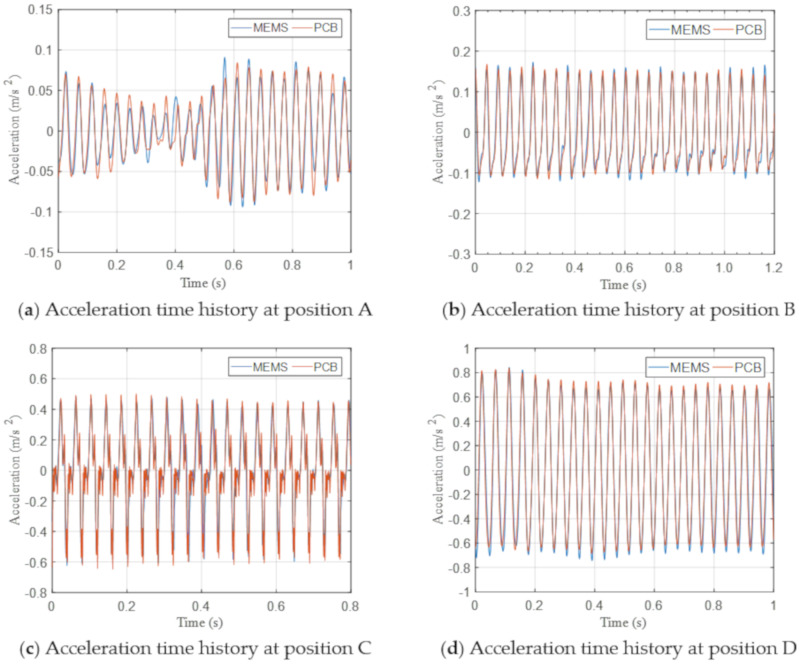
Measured acceleration time histories at different locations.

Figure 16 shows a comparison of the measured acceleration time histories collected by two different sensing systems at four measurement points (i.e., positions A–D), wherein the plan distance decreases from position A to position D. The sensors near the sheet piling measured considerably larger vibration levels. The measurement results from two sensing systems were generally consistent without large deviation. The consistent results shown in Figure 16a indicate that the IoT sensing system can measure vibration as low as 0.05 m/s^2^. The agreement between two signals proves the ability of the IoT sensing system to measure vibrations of various amplitudes accurately in practical site conditions.

**Figure 17:**
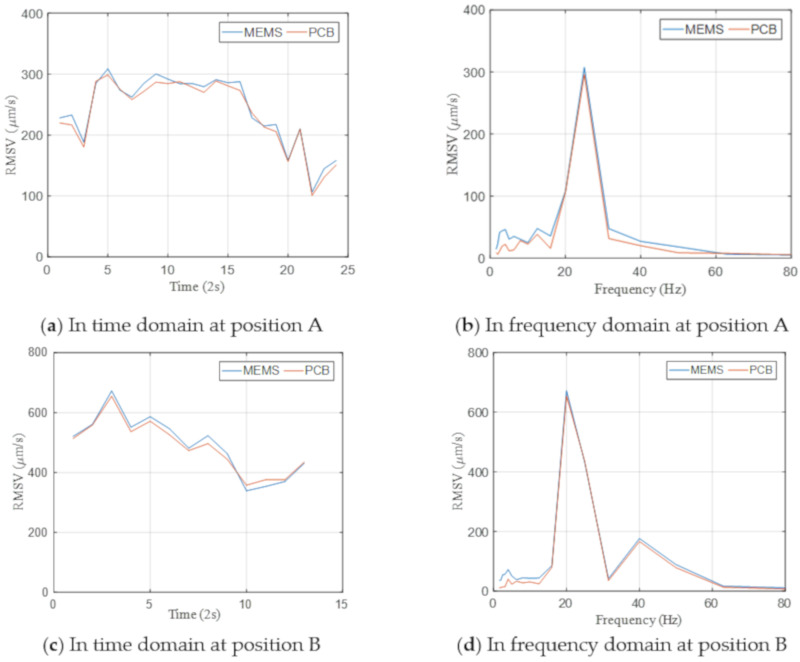

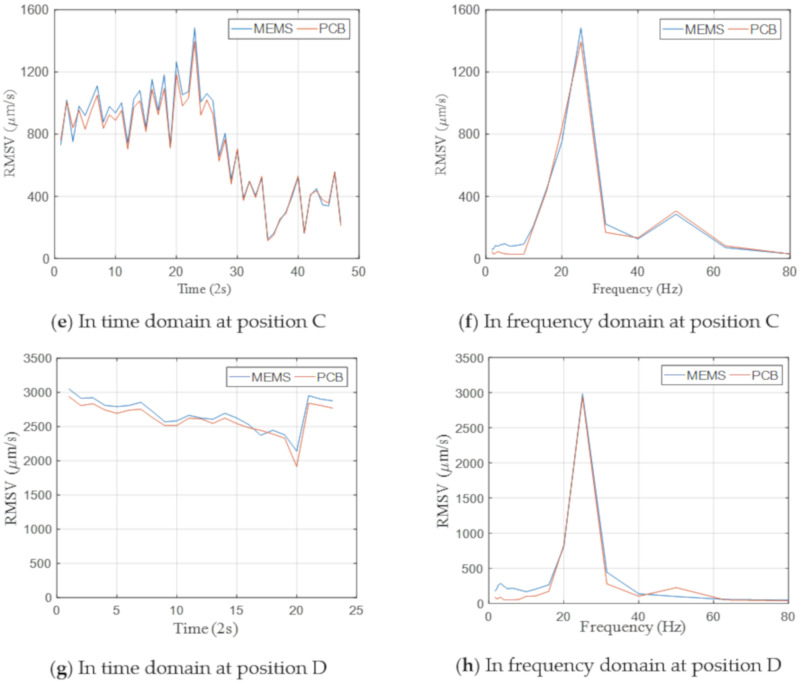
RMS velocity in the 1/3 octave band in time and frequency domain.

**Figure 18:**
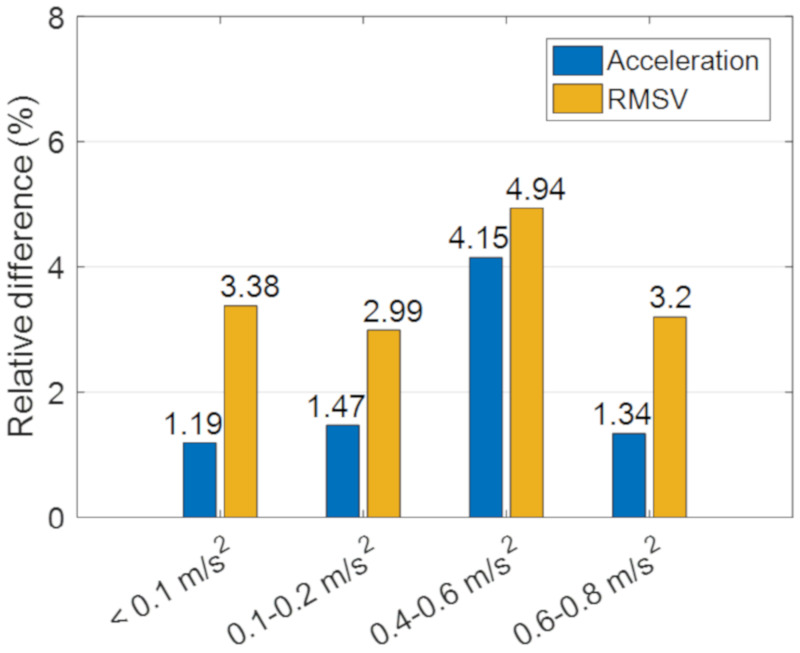
Relative differences of acceleration and RMS velocity in the 1/3 octave band between the MEMS and PCB accelerometers.

As shown in Figure 17, the RMS velocity in the 1/3 octave band in both time and frequency domain were also calculated in real time by the Raspberry Pi in the IoT sensing system and by the computer in the wired system. The results from the two monitoring systems agree with each other satisfactorily. The relative differences presented in Figure 18 are generally less than 5%. The RMS velocities in the 1/3 octave band at a far point (i.e., position A) are lower than 300 μm/s, which satisfies the threshold for office buildings as suggested by the ASHRAE [13]. While the RMS velocities in the 1/3 octave band reach up to 3000 μm/s at a near point (i.e., position D), and the corresponding PPV is around 5 mm/s, which is lower than the PPV limit of 7.5 mm/s required by the Code of Practice for Foundations in Hong Kong [11] for protecting nearby structures. Therefore, the vibration amplitude induced by the sheet piling was regarded acceptable and the alarm signals were not triggered in the field tests.

## 5. Summary and Discussion

This study presents the design, development, and validation of a wireless IoT sensing system for real-time construction-induced vibration monitoring and impact assessment. The hardware of the developed IoT sensing system consists of the Raspberry Pi board, MEMS accelerometer, 4G Internet dongle, and solar battery. The Raspberry Pi is used to collect and analyze digital signals measured by the MEMS accelerometer. Local signal processing, including data filter, integration, and evaluation of real-time vibration levels with respect to PPV, RMS velocity in 1/3 octave band, and RMS acceleration in 1/3 octave band, are realized in the Raspberry Pi. The raw and processed data, as well as the analyzed results are transmitted to the MySQL database on the public cloud through the 4G mobile network in a timely manner. Solar panels with a rechargeable battery are used as a continuous power supply. The operation software and NTP time synchronization mechanism are developed accordingly. The database and alarm functions are implemented in the cloud system. In addition, a webpage-based user interface is developed to display real-time measurement results and alarm signals, which can be accessed remotely from the authorized computer or mobile devices via the Internet. The salient features of the IoT vibration sensing system include: (1) small size and standalone wireless sensor nodes that enable easy installation without any cabling work, (2) low hardware costs and low power consumption, (3) independent sensor nodes avoiding hierarchic sensor network, (4) reliable data transmission without data loss, (5) NTP time synchronizing allowing high-precision data sampling, and (6) real-time data processing, vibration assessment, and alarm capability.

The accuracy of the selected MEMS accelerometer (LSM9DS1) has been verified through the comparison with the traditionally wired accelerometer in the laboratory experiments. Moreover, the IoT vibration sensing system was tested on a real constriction site in Hong Kong with ongoing sheet piling construction work. Both the laboratory and field experimental results indicated that the developed IoT sensing system can measure construction-induced vibrations with satisfactory accuracy in both time and frequency domain. The computation power of the Raspberry Pi and cloud enables the real-time computation of different vibration indicators and assess the vibration impact by comparing various prespecified vibration limits. This IoT vibration sensing system will be soon implemented in another construction site in Hong Kong for long-term construction vibration impact monitoring and assessment. 

Notably, the Raspberry Pi is an open-source platform that enables connections to various types of sensors with compatible ports. Therefore, the developed IoT sensing system can be conveniently extended to other monitoring functions, such as MEMS microphones for noise measurement, temperature and humidity sensors for environmental monitoring, ultrasonic sensors for damage detection, and higher sensitivity vibration sensors if necessary.

## Figures and Tables

**Table 1:** Resolutions of LSM9DS1 MEMS accelerometer corresponding to various measurement ranges.

Range	Resolution (mg/LSB)
±2 g, 16-bit mode	0.061
±4 g, 16-bit mode	0.122
±8 g, 16-bit mode	0.244
±16 g, 16-bit mode	0.732

**Table 2:** Characteristics of HUAWEI USB 4G dongle.

Parameters	Values or Description
Data Transfer Rate (LTE)	DL:150 Mbps/UL:50 Mbps
Supply Voltage	4.75 V to 5.25 V
Power Consumption	800 mA (theoretical)
Model Size	94 mm × 30 mm × 14 mm
Weight	35 g

**Table 3:** Register mapping for LSM9DS1 accelerometer.

Name	Description	Address
OUT_X_L_XL	Output of accelerometer’s *X*-axis low event	0×28
OUT_X_H_XL	Output of accelerometer’s *X*-axis high event	0×29
OUT_Y_L_XL	Output of accelerometer’s *Y*-axis low event	0×2A
OUT_Y_H_XL	Output of accelerometer’s *Y*-axis high event	0×2B
OUT_Z_L_XL	Output of accelerometer’s *Z*-axis low event	0×2C
OUT_Z_H_XL	Output of accelerometer’s *Z*-axis high event	0×2D

**Table 4:** System cost comparison.

Component	Commercial System	IoT Sensing System
Sensor	US $1425	US $15
DAQ	US $970	US $55
Cable	US $225	/
Sum	US $2620	US $70

## References

[1] Athanasopoulos G., Pelekis P. (2000). Ground vibrations from sheetpile driving in urban environment: Measurements, analysis and effects on buildings and occupants. Soil Dyn. Earthq. Eng..

[2] Hiller D., Hope V. (1998). Groundborne vibration generated by mechanized construction activities. Proc. Inst. Civ. Eng. Geotech. Eng..

[3] Hwang J.-H., Tu T.-Y. (2002). Ground vibration during gravel pile construction. J. Mar. Sci. Technol..

[4] Kim D.-S., Lee J.-S. (2000). Propagation and attenuation characteristics of various ground vibrations. Soil Dyn. Earthq. Eng..

[5] Tavasoli O., Ghazavi M. (2018). Wave propagation and ground vibrations due to non-uniform cross-sections piles driving. Comput. Geotech..

[6] Zhu S., Shi X., Leung R.C., Cheng L., Ng S., Zhang X., Wang Y. (2014). Impact of construction-induced vibration on vibration-sensitive medical equipment: A case study. Adv. Struct. Eng..

[7] International Organization for Standardization (1997). ISO 2631-1: 1997. Mechanical Vibration and Shock-Evaluation of Human Exposure to Whole-Body Vibration-Part 1: General Requirements.

[8] British Standards Institution (1993). BS 7385-2: 1993. Evaluation and Measurement for Vibration in Buildings: Guide to Damage Levels from Groundborne Vibration.

[9] British Standards Institution (1992). BS 8228-4: 1992. 5228: Part 4: 1992 Noise Control on Construction and Open Sites Part 4: Code of Practice for Noise and Vibration Control Applicable to Piling Operations.

[10] European Committee for Standardization (1993). ENV 1993-5. Eurocode 3: Design of Steel Structures—Part 5: Piling.

[11] Building Department (2017). HKSARG 2017. Code of Practice for Foundations 2017.

[12] Ministry of Ecology and Environment (2017). HJ 918-2017. Technical Specifications for Environmental Vibration Monitoring.

[13] ASHRAE Handbook (2011). Heating, Ventilating, and Air-Conditioning Applications.

[14] New B.M. (1986). Ground Vibration Caused by Civil Engineering Works.

[15] Head J.M., Jardine F.M. (1992). Ground-Borne Vibrations Arising from Piling.

[16] U.S. Department of Labor (1926). OSHA Reorganization Plan, No. 14 for 1950. Safety and Health Regulations for Construction.

[17] California Department of Transportation (2013). Transportation and Construction-Induced Vibration Guidance Manual.

[18] Civil Engineering and Development Department (2019). Guidance Note, No. GN 10 Vibration Monitoring. Mines Division GEO.

[19] Building Department (2009). HKSARG 2009. Code of Practice for Site Supervision.

[20] Wong K.-Y. (2007). Design of a structural health monitoring system for long-span bridges. Struct. Infrastruct. Eng..

[21] Zhou H., Ni Y., Ko J. (2008). A data processing and analysis system for the instrumented suspension Jiangyin Bridge. World Forum on Smart Materials and Smart Structures Technology. Proceedings of the SMSST’07, World Forum on Smart Materials and Smart Structures Technology (SMSST’07).

[22] Ni Y., Xia Y., Liao W., Ko J. (2009). Technology innovation in developing the structural health monitoring system for Guangzhou New TV Tower. Struct. Control Health Monit..

[23] Xu Y., Zhan S. (2001). Field measurements of Di Wang tower during typhoon York. J. Wind Eng. Ind. Aerodyn..

[24] Straser E.G., Kiremidjian A.S., Meng T.H. (2001). Modular, Wireless Damage Monitoring System for Structures. U.S. Patent.

[25] Lynch J.P. (2002). Decentralization of Wireless Monitoring and Control Technologies for Smart Civil Structures.

[26] Wang Y., Lynch J.P., Law K.H. Wireless structural sensors using reliable communication protocols for data acquisition and interrogation. Proceedings of the 23rd International Modal Analysis Conference (IMAC XXIII) 2005.

[27] Rice J.A., Spencer B. (2008). Structural Health Monitoring Sensor Development for the Imote2 Platform. Sensors and Smart Structures Technologies for Civil, Mechanical, and Aerospace Systems 2008.

[28] Jo H., Sim S.-H., Nagayama T., Spencer B. (2012). Development and application of high-sensitivity wireless smart sensors for decentralized stochastic modal identification. J. Eng. Mech..

[29] Zou Z., Nagayama T., Fujino Y. (2014). Efficient multihop communication for static wireless sensor networks in the application to civil infrastructure monitoring. Struct. Control Health Monit..

[30] Chae M., Yoo H., Kim J., Cho M. (2012). Development of a wireless sensor network system for suspension bridge health monitoring. Autom. Constr..

[31] Kohler M.D., Hao S., Mishra N., Govinda R., Nigbor R. (2015). ShakeNet: A Portable Wireless Sensor Network for Instrumenting Large Civil Structures.

[32] Sazonov E., Li H., Curry D., Pillay P. (2009). Self-powered sensors for monitoring of highway bridges. IEEE Sens. J..

[33] Hu X., Wang B., Ji H. (2013). A wireless sensor network-based structural health monitoring system for highway bridges. Comput. Aided Civ. Infrastruct. Eng..

[34] Liu Z., Yu Y., Liu G., Wang J., Mao X. (2014). Design of a wireless measurement system based on WSNs for large bridges. Measurement.

[35] Ni Y., Li B., Lam K., Zhu D., Wang Y., Lynch J., Law K.H. (2011). In-construction vibration monitoring of a super-tall structure using a long-range wireless sensing system. Smart Struct. Syst..

[36] Mechitov K., Kim W., Agha G., Nagayama T. High-frequency distributed sensing for structure monitoring. Proceedings of the First International Workshop on Networked Sensing Systems.

[37] Perahia E., Stacey R. (2013). Next Generation Wireless LANs: 802.11 n and 802.11 ac[M].

[38] Lee J.S., Su Y.W., Shen C.C. A Comparative Study of Wireless Protocols: Bluetooth, UWB, ZigBee, and Wi-Fi. Proceedings of the 33rd Annual Conference of the IEEE Industrial Electronics Society (IECON).

[39] Jason M. 3 Key Factors That Determine the Range of Bluetooth. www.bluetooth.com/blog/3-key-factors-that-determinethe-range-of-bluetooth.html.

[40] Gubbi J., Buyya R., Marusic S., Palaniswami M. (2013). Internet of Things (IoT): A vision, architectural elements, and future directions. Future Gener. Comput. Syst..

[41] Abdelgawad A., Yelamarthi K. (2017). Internet of things (IoT) platform for structure health monitoring. Wirel. Commun. Mob. Comput..

[42] Chandrasekaran S., Chithambaram T., Khader S.A. (2016). Structural health monitoring of offshore structures using wireless sensor networking under operational and environmental variability. Int. J. Environ. Ecol. Eng..

[43] Mahmud M.A., Bates K., Wood T., Abdelgawad A., Yelamarthi K. A complete internet of things (IoT) platform for structural health monitoring (shm). Proceedings of the 2018 IEEE 4th World Forum on Internet of Things (WF-IoT).

[44] Liu Q., Ma Y., Alhussein M., Zhang Y., Peng L. (2016). Green data center with IoT sensing and cloud-assisted smart temperature control system. Comput. Netw..

[45] Pereira R.I., Dupont I.M., Carvalho P.C., Jucá S.C. (2018). IoT embedded linux system based on Raspberry Pi applied to real-time cloud monitoring of a decentralized photovoltaic plant. Measurement.

[46] Piyare R., Lee S.R. (2013). Towards internet of things (iots): Integration of wireless sensor network to cloud services for data collection and sharing. arXiv.

